# A Critical Analysis of the “Review” of Professor B. W. Dudley’s Article on Aneurism, in the November No. of the Louisville Medical Journal

**Published:** 1850-02

**Authors:** 


### REVIEWS.

Art. III.—*A Critical Analysis of the "Review" of Prof. B. W. Dudley's article on Aneurism, in the November No. of the Louisville Medical Journal.* Transylvania Medical Journal, Vol. 1, No. III.

Gentle reader, we have been analyzed—not skilfully by the practiced hand of an experienced manipulator—but rudely, by one who raves as if he thought,

"On such a theme 't were impious to be calm."

Doubtless, as the eye of the editor of the Transylvania Journal of Medicine traced the foul characters that soiled the virgin purity of the sheet before him, he gloated over the work of destruction which he vainly fancied was being accomplished. With what demoniacal satisfaction he contemplates the mangled remains of his pigmy victim, torn limb from limb, and strewed in confusion around him. We are happy to inform our readers, however, that a sufficient amount of vitality remained, to reanimate the scattered fragments; and we once more stand erect before them, prepared to do battle in the cause of truth, reason, and common sense.

We assure our readers that the tone of the analysis, better suited to the pot-house politician than to the dignity of a medical professor, is foreign to the ordinary bearing of its author, who has been, doubtless, betrayed into ungovernable passion by deep devotion to one to whom he is indissolubly bound by ties of blood and long years of unbroken association. But we have hope of him

—passion will subside, and calm reason again resume her legitimate supremacy:

> "Those hearts that start at once into a blaze,
> And open all their rage, like summer storms
> At once discharged, grow cool again and calm."

We remember no instance in which true science has been advanced by grandiloquent declamation, opprobrious epithets, or personal defamation; we, therefore, decline to follow the young professor in his "revels in the filthy stew of detraction," "personal malevolence," "splenetic assaults," "villification," etc., etc.

As, in some minds, we often observe strange admixtures of exalted virtue and debasing vice, pure and refined sentiment linked to the lowest and most grovelling propensities, so, in the analysis before us, we discover allegations of the basest motives, epithets the most foul, and personal detraction the most unmerited, strangely associated with the purest and most lofty sentiments that can adorn the human character. We will be charitable to the analyst, and believe that his indiscretion is the result of a sudden ebullition of passion, and, hoping that his fine poetic taste may extract a lesson of wisdom that will serve him well in future, we commend to his serious consideration a truth, which does not seem to have exerted on him its due weight:

> "When anger rushes unrestrained to action,
> Like a hot steed it stumbles in its way:
> The man of thought strikes deepest, and strikes safest."

We repel, with "unutterable scorn," the charge of "personal malevolence," in our review of Professor Dudley, and the chief object we have in view, in noticing the analysis, is to place ourself right before the readers of this Journal. He who writes at all, is extremely fortunate if he does not, sometimes, let fall expressions which, after mature deliberation, he would gladly alter, amend, or wholly omit; but if such defects be justly chargeable to our review of Prof. D., we have not been able to discover them, but lest others may think otherwise, we declare, that no personal disrespect was intended. Our allusions were exclusively directed to the dogmas and claims of Prof. D., and touched his standing only as a teacher and writer.

We feel confident that we have not transcended the limits observed by many of the best critics in medicine—and that, while our strictures are confined to opinions, and do not degenerate into personalities, the utmost freedom will not only be tolerated, but justified and approved by the unanimous voice of the profession. Several examples occur to our recollection at this moment, to which we beg to call the attention of our analyst. One of these we give, on account of the known purity of heart, kindliness of feeling, unadulterated good taste, and high standing of its author; the others are from the colleagues of our analyst, with whom he is in daily association.

Prof. Bartlett is justly renowned for his good taste and gentlemanly bearing toward members of the profession, without the slightest tincture of gall in his nature; and, for ourself, we feel no apprehension of giving just cause of offence, while circumscribed by the principles and bounds of criticism observed by that truly great and good man. Note Professor Bartlett's strictures on the doctrines of the venerable Rush, and the analyst will scarcely venture to rank his distinguished kinsman higher in point of fame or ability than that wonderful man—and beside Prof. Bartlett's strictures, our notice of Prof. D. will assume as mild an aspect as his best friends can desire. Speaking of the leading feature of Dr. Rush's doctrine of fever, Dr. B. says: "It is not merely abstract, gratuitous, and unintelligible, but in direct and manifest opposition to all common sense, to all true philosophy, and to all correct observation. * * * * It may be safely said, I think, that in the whole vast compass of medical literature, there cannot be found an equal number of pages, containing an equal amount and variety of utter nonsense and unqualified absurdity,—a more heterogeneous and ill-adjusted an assemblage, not merely of unsupported, but of unintelligible and preposterous assertions, than are embodied in this exposition of this theory."*

Had Prof. Bartlett any selfish purpose to subserve?—Was he seeking the gratification of a revenge incited by disappointment? Did he intend any personal disrespect, or aim to fix a stigma on the memory of the great Rush? No! He was nobly battling for truth and correct philosophy, and while he pitilessly struck down the false theories, he not the less venerated the man, and paid due homage to his well deserved merits, declaring, "notwithstanding all this, Dr. Rush was an honor and an ornament to his country."

Has the analyst seen the review of this same "Philosophy of Medical Science," from the pen of his colleague, Prof. Annan? We regret that we cannot lay our hand on the Southern Literary Messenger that contains it, as it would afford us pleasure to cull some of its beauties and exhibit to the analyst how far Prof. A. thinks the bounds of legitimate criticism extend. Was Prof. A. actuated by "that ruffian-like spirit which seeks pleasure in wilful and oblique aggression, and revels in the filthy stew of detraction?" Had Dr. B. interfered with the prospects of Dr. A., or did the latter intend only to express the opinions he entertained of the doctrines of the former, with no purpose to give personal offence? We are bound to believe that no disrespect was intended, and that Prof. B. did not regard Prof. A. as overleaping the recognized limits of justifiable criticism, as these gentle-

* Philosophy of Medical Science, p. 225.

men were afterwards associated together in the same school, and met as gentlemen and friends.

Has the analyst seen the review of Dr. Lewis's "Medical History of Alabama," by his present colleague, Prof. Boling? Does he think that Prof. B.'s strictures were prompted by selfish and unworthy motives, or that he was actuated by the laudable desire to refute error and to establish truth?

Has the analyst met with the review of Dr. Caldwell's "Physiology Vindicated," by Prof. Peter? Unusual severity is apparent in every line, and yet we will not charge, nor do we believe that the analyst will charge that severity to "rude and intemperate feeling," "malevolence," or "scribbling impertinence."

A single paragraph is so appropriate that we extract and adopt it, as an ample apology for the manner in which we have discharged the disagreeable task we have undertaken.

We quote Prof. Peter, only substituting the name of Prof. Dudley for that of Dr. Caldwell:

"Truth is more venerable, exalted, and powerful than the Professor, and error must be continually unmasked, to prevent her from imposing herself upon those who have not the time, inclination, or ability to look beneath the surface of things. The respect which I, in common with many others, entertain for the venerable Prof. Dudley, cannot excuse me for the desertion of her standard; and the apparent want of courtesy, which may be supposed to exist in my animadversions, must be excused in the necessity of the case. Truth must be promulgated and sustained; and, if those who oppose it are offended, the fault is only with themselves."*

---

* Review of Dr. Caldwell's Pamphlet, entitled "Physiology Vindicated," p. 4.

The truth is, the reviewer may often be severe in regard to opinions and doctrines, without rendering himself fairly liable to the imputation of base motives or personal disrespect to his author. We should have varied in nothing our critique of Prof. D., if he had been, to us, an entire stranger. The young professor ought to learn in the outset of his career as caterer for the medical public, and we have alluded to the examples above to show, that there is very generally recognized a proper and clear distinction between men and authors—between persons and opinions; with the former, we have naught to do, and we would not parade the personal faults of an author before the public, if we knew any to exist, and it were in our power to array them in solid phalanx to overwhelm with confusion. Our concern is with the mental offspring, and we beg the poor privilege of dealing with the bantlings as we may think they merit by their comeliness or deformity.

Our strictures on Prof. D. were not more stringent, or less liberal, than those daily uttered by him on the labors of others. How many of his lectures, during every session, are introduced by the regret that he stands alone, and that he does not know a single author to whom he can refer, that has treated the subject in a rational manner—that "the books are all wrong," has been with him a stereotyped expression, for a score of years; and we have heard more than one member of the present class declare that his lectures were well calculated to destroy all faith in medical records, and to discourage the student from the drudgery of laboring through what is uniformly represented to him as an unbroken series of error. But, perhaps, age may confer a right that is denied to juniors.

We are charged with injustice to Prof. D., and that injustice is ascribed to the base feeling of revenge, excited by disappointed ambition. We have never applied for a place in Transylvania, and scorn the unjust imputation.

The time was, when the tallest intellects that adorn the profession, sought, without derogation, that high honor; but the system of nepotism, which has so long ruled the destiny of Transylvania, has caused those days of glory to be numbered with the things that were.

With how much more force might we retort on the analyst, that his zeal is inspired by obligations he owes his kinsman for the place he occupies, and that regardless of truth, honor, or reason, he is bound to follow the lead of his patron. But we prefer to be charitable, if not just, and to judge others by our own feelings; conscious of no unworthy motive in our own breast, we will not believe that he is actuated by a mercenary spirit, unbecoming a man of truth and a gentleman. We would rather impute his untempered zeal, and rash efforts at defence, to a firm and sincere, but blind, faith in the dogmas taught by his uncle—a faith, naturally fanned to greater intensity by gratitude for kindnesses and favors bestowed without measure.

As to the young professor's vulgar allusion, in regard to the supposed effect of our introduction into the school, we may say that we should have been truly mortified if we had accepted an appointment, and our acceptance had been followed by as rapid and steady a decline as that which succeeded the induction of the analyst into the chair of pathological anatomy. Each succeeding class, since his appointment, has grown "small by degrees and beautifully less," until a respectable class of over two hundred members has dwindled to little over one-fourth of that number. The difference between the two opinions entertained on this subject, is as obvious as that which exists between hypothesis and demonstration. He *thinks* that our connection with the "old patient" would have proved fatal; we *know* that his has resulted in utter exhaustion, and brought the poor sufferer to the last extremity. That we are not singular in our opinion is quite evident, since the analyst and his associates have, in despair, proposed the last desperate remedy—a change of climate. Who knows but a winter residence on the banks of "*la belle riviére*," and a summer's sojourn in the "Athens of the West," may result in the restoration of the "old patient" to pristine health and vigor?

Seriously, no one can feel more sincere regret for the rapid decline of Transylvania than ourself. In our opinion various causes have conspired with that system of favoritism that disregards qualification, to effect this lamentable result. Some of these causes were unavoidable, and others may yet be removed. The facilities for intercommunication by means of steam power have produced a decided tendency to centralization in the large cities, in matters of education, as well as in commerce—and hence, the rapid decline of interior schools, and the extraordinary growth of those more favorably located.—This result has been materially promoted, by the superior advantages for anatomical and clinical instruction, offered in the midst of a dense and numerous population.

But the most fatal blow to Transylvania comes, very strangely, from her own Professors, who have sought, and are now seeking, to remove the school to another and a distant city, thereby admitting that, in their judgement, Lexington is not a suitable site for such an institution, and thus giving the vantage ground to the enemies of the school, who are anxiously seeking its annihilation.

Let a united faculty exert all its energies to foster and sustain the school where it has flourished so many years, and frown indignantly on every intrigue for its removal, and, with the ample means for instruction in an extensive and valuable library; in the costly apparatus and well filled museum, which the extraordinary liberality of the people of Lexington has placed at command, and a respectable school will be ensured as long as medical science continues to be cultivated. Dismiss the delusive hope of successful competition with more favored neighbors, and banish petty jealousy from the mind, and a reasonable expectation may be indulged, not of a full restoration of Transylvania to her former proud distinction, but, that she may attain a standing quite as elevated as any interior school in the country. This, we believe, the present faculty may accomplish if they choose to pursue a wise and prudent course. To hope to effect more would be as utopean as to expect Lexington to rival New York in commerce.

Another cause, and not an insignificant one, for the hostility displayed against the institution is to be found in the overbearing, dictatorial and abusive spirit so prominently exhibited in the analysis before us. All who have dared to dissent from any of the dogmas of Professor Dudley, have been ruthlessly assailed, and their usefulness and professional standing destroyed, as far as it was in the power of his too ardent and over zealous friends and defendants to accomplish the work; such may also be our fate, but, if so, we shall go down with the conviction that we have faithfully discharged our duty, and cheered by the consolation that the right will sooner or later triumph:

> "Truth crushed to earth, shall rise again—
> The eternal years of God are hers;
> But error, wounded, writhes with pain,
> And dies amidst its worshippers."

Admitting Professor D.'s superior attainments in surgery, we believe we are still in a better position, than the analyst, to form a true estimate of the professional merits of Professor D. Personally friendly, we have no prejudice to blind; no malignant feelings to gratify; bound to him by no unusual ties, we have no bias to mislead—no obligations to cause us to falter in the advocacy of truth.

We respect Professor Dudly as an able—an extraordinary man—a man whom nature has endowed with transcendent abilities, and an indomitable energy and perseverance. As a teacher of anatomy, we do not believe he has an equal; we doubt, if a century produces a superior. Here, in fact, was his power, and the secret of his fame as a teacher, a fame, that waned from the first moment he ceased to teach facts, and undertook to impart principles. As an operative surgeon, he also stands, in our estimation, preëminent. Great, however, as we esteem him, we believe that with a little less self reliance, he would have accomplished a far brighter and more enduring reputation than that which he has achieved, or can hope hereafter to achieve. Less pride of opinion, and more distrust of self, would have induced a more candid and careful analysis of the doctrines of others; enlarged and rendered more perfect his own views; and softened much of the harsh judgment which he daily pronounces on the labors of his predecessors and contemporaries.

With a brief notice of some of the points in the analysis, more worthy of consideration than those we have passed in review, we shall dismiss the subject, with the determination to pursue, hereafter, the original purpose of confining our attention to such essays as may be published by Professor D.; leaving the field clear to such volunteers as may enter it (we presume) with the impression that the Professor is unable to defend and maintain his own position. The analyist introduces his critical remarks with the request, that "it be remembered that the essay of Professor Dudly was designed as a plain and concise statement of his own experience—that he designedly avoided detail," etc. This is a virtual acknowledgment of the short-coming of the essay, which we had the honor to review, for, in the prospectus of the Transylvania Medical Journal, it was announced that those essays would "present a *full* record of his (Professor D.'s) extensive experience," now, however, we are told that detail is purposely avoided, and that we are to have only an imperfect synopsis—a crayon sketch instead of a perfect picture. Well, we will bear this in mind hereafter, and lower, somewhat, the standard by which we intended to measure the merits of the successive numbers of the series. Indeed this is not the first evidence that the volunteer champion of Professor D. has given, that he thought the essay neither so perfect or lucid as was desirable; else, why did he deem it necessary to analyse, re-arrange, and systamatise it in the second number of his journal? This was required by the imperfection or obscurity of the essay itself, and its unintelligible character is thus established by the editor's own act; or, he has paid his indulgent readers the poor compliment, to suppose that they could not fathom the depths of the original essay, and extract from its recesses the excellencies and beauties with which he would fain *force* us to believe it is enriched.

The analyst occupies, very unnecessarily, nearly two pages to prove what we very clearly admitted; that many of the ablest surgeons agree in "attributing to Mr. Hunter the honor of first suggesting the most successful mode of treating aneurism." We pronounced this a "common error," and gave, from original authority, the evidence on which our opinion was founded. The analyst labors hard to shield Professor D. behind the great name of John Hunter, and would fain make the impression that we have treated that distinguished man with injustice, and that Professor D. occupies the position of his defender. We are pleased to hear that Professor D. speaks well of at least one surgical writer, for it was not his habit to treat John Hunter or any other author with much respect when we had the honor of listening to his lectures. But this is a mere ruse and not an unusual course with partizans when foiled by facts, and in want of argument. The hope of escape lies only in distracting the mind by making a diversion on points not at all material, and only appearing incidentally, while the matter truly at issue is passed over so lightly as scarcely to attract attention. John Hunter requires no eulogy from us, nor can the analyst, with all his oratory, add one mite to a reputation as broad as the bounds of science, and as enduring as time itself. The analyst declares that Professor D. had learned, from the mature teachings of his predecessors, "that such treatment (by compression) had proven so painful, hazardous, and inefficacious, as to be condemned and discredited, although a few cases of success had been recorded."

Now, we believe, that we are borne out by authentic history, in asserting that compression has been resorted to in the treatment of aneurism, since the sixteenth century, when it was tested, and found to be a valuable means, by John of Vigo, Paré, Hildanus, etc. About the middle of the seventeenth century, the tourniquet was put in requisition, by many surgeons, to check the flow of blood into the aneurismal sac, and various contrivances were adopted to effect suitable pressure on the tumour itself.

The practical importance of this subject will excuse us for giving the following extract, pointing out, as it does, in a clear and concise manner, many of the circumstances that should control us in the use of compression, and the character of cases in which we may reasonably hope for success. At the same time, it will sustain the position, we have assumed, that the remedy is neither novel nor of universal application. Professor D.'s first case was treated by compression in 1814, the extract dates in 1807:

"In order to obtain a completely radical cure of aneurism, by means of compression, it is necessary that there be a certain degree of vitality in the proper coats of the artery, that the pressure be sufficient to keep them in firm contact, and excite in them the due degree of adhesive inflammation. It is, therefore, more likely to fail when the disease has been produced by internal causes, where a considerable portion of the artery is disorganized above the laceration or rupture, and not disposed to undergo the adhesive inflammation. *Some surgeons think that the compression ought to be employed in every case of aneurism;* but experience has shown, that it proves injurious in all those which are of long standing, of a large size, painful, and false, as they were formerly called; when the pressure is applied below the place of the injury in the artery; when, on account of the size and exqisite sensibility of the aneurism, and the depth of its situation, and the swelling of the contiguous parts, a sufficient degree of pressure cannot be employed to keep the sides of the artery in firm and steady contact; and, lastly, in all cases, where, from the history of the disease, we have reason to suppose, that it has arisen from a steatomatous or earthy degeneration of the artery. In cases the reverse of these just mentioned, compression has often effected a complete cure; of course it is a method which ought not to be entirely excluded from practice."*

We believe that our readers will find more available information in this short extract, in regard to the nature of pressure in the treatment of aneurism, and the peculiar cases to which it is adapted, than can be extracted from the whole of Professor D.'s essay and all that has been added by his defender.

Dr. Parish in 1810, four years prior to Professor D.'s first case, remarks, after alluding to Scarpa's commendation of the bandage in certain cases of aneurism: "Other surgeons have used compression with a pad and tourniquet, and suddenly stopped the circulation in the tumor. This treatment is then continued until the limb becomes edematous, when the tourniquet is directed to be remov-

---

*Ed. Med. and Sur. Jour., v. 3, p. 489—Review of Scarpa on Aneurism.

ed; and the pressure of a pad and roller is said to be afterwards sufficient."*

We find in the *Archives Génerales de Medicine*,† an allusion to an article by Mr. Giraldés, on the treatment of popliteal aneurism by compression. In view of the serious accidents that not unfrequently follow the ordinary operation, he inquires if these may not be avoided, and a radical cure effected, by means of compression. It is then remarked: "L'auteur rappelle d'abord que cette methode a été employée avec succès par Dubois, White, Viricel, et Dupuytren. Plusieurs chirurgiens irlandais l'ont mise en usage dans ces derniers temps avec non moins succès; l'auteur rapporte ici les faits publiés par MM. Hutton, Cusack, Bellingham, Allen et Grentrex. MM. Liston et Bellingham se sont encore servis de la compression pour deux cas d'anevrysme femoral, et ont également reussi. Voila donc huit observations nouvelles qui, ajoutées a celles des années précédente, sont en faveur de la compression."

In the American Journal of the Medical Sciences, for January, 1847, we have a table extracted from The Dublin Quarterly Journal, from which it appears "that twenty-nine cases of aneurism—six femoral and twenty-three popliteal—have been treated by pressure upon the artery leading to the sac; of which number, nineteen occurred in Dublin; and that in four, the femoral artery was tied, chiefly from want of confidence in pressure, on the part of either surgeon or patient, and that in twenty-five instances this mode of treatment was successful."‡

We are fully aware that the mode of application pro-

---

*Eclectic Repertory, v. 1, 509.

†Tom. 8 p. 104.

‡Amer. Jour., No. xxv, p. 186. If our readers desire further information on this subject, they may consult with advantage the same journal, Nos. 22, 26, 31, 35, 36.

posed by Professor D. differs from that of Mr. Bellingham, but the principle is the same, and the cases therefore in point—the principle is undying; the mode does, and ought to vary with circumstances.

We have provoked mortal offence by intimating the possibility that Professor D. might have mistaken a functional disorder of the heart for an organic lesion of that organ. Well, we have known the Professor to fall into such errors; and, we presume, they have occurred to the ablest men that ever adorned the profession; and serve rather to illustrate the imperfection of our means of diagnosis, in all the Protean forms of disease, than to detract from the fame or ability of the careful practitioner.

Corvisart, after repeated and most deliberate examinations, pronounced a patient to be the subject of *aneurism of the heart*, and this diagnosis was concurred in by several other physicians who were consulted, and yet the autopsy subsequently revealed the fact, that *all the thoracic organs were perfectly sound*, and that the patient had succumbed to *disease of the colon*. The reviewer, evidently, did not consider a similar error wonderful or materially derogatory to his character as a diagnostician, since he very candidly makes the following confession in a note to this case:

"At this moment there stands before our window, a healthy man, whom, many years ago, I gave up, as the victim of active dilatation of the heart, and who was subsequently cured, to our astonishment, by the aloes, spirit of ammonia, and bitters, of a sagacious empyric."*

On the other hand, aneurisms of the heart and large arteries, are doubtless often overlooked. Who does not remember the case of the lamented Liston, in whose body the examination post mortem, revealed an aneurism of the aorta as large as an orange; yet, during life Drs.

*Med. Chi. Rev., 1823, p. 469.

Watson and Forbes could detect no such organic lesion, and refused to admit its existence even when suggested by the sufferer?

The truth is, errors in diagnosis occur to all, and do not constitute any just cause of reproach, when the practitioner has conducted his investigations with care, commensurate with the importance of the disease, and aided by all the means at the command of the profession.

These errors of the truly eminent, however, render us exceedingly cautious in receiving extraordinary cases and wonderful cures, unaccompanied by such details, as will place us in a position to verify the correctness of the diagnosis; hence, we suggested that the report of Professor D.'s cases was too meager, to serve as a foundation for any practice—without details, the practice would be based on the *opinions* of the writer—with them, on *facts*.

On the subject of the bandage, as a remedial agent, we have little to say at present, having already exceeded our limits. We shall, doubtless, have occasion to allude to it frequently in the course of our strictures on the forthcoming essays of Professor D. We may say here, however, that, as far back as Galen, very particular directions were given for the application of the bandage in a great variety of diseases, and students were urgently pressed to practice its adjustment to the various parts of the sound body, or to a figure constructed for that purpose, in order that they might thus become expert in the use of an agent, for which they would have daily demands. For an excellent ancient account of the bandage, the material of which it should be composed, its application to "the treatment of wounds, fractures, luxations, amputations, and the like," we may refer the reader to Hiester,* who

*Gen. Sys. Sur., p. 262.

wrote early in the past century, in whose work may be found the importance of the bandage fully appreciated, and very minute practical instruction in regard to it given. He says: "they (bandages) are often medicines of themselves, being the sole application for the cure of the disorder; as in many fractures, luxations, hemorrhages," etc.

But Hiester does not pretend to be a leader in this matter, but refers to several elaborate works on the same subject, among which are those of Galen, Verduc, Le Clerc, etc.

We have no reply to offer to the unique and able argument of the analyst in support of that novel principle, "that inefficient pressure increases hemorrhage." What think our readers are these arguments? "Facetious critic," "ignorant, pert coxcomb," "most sapient reviewer," etc.; constitute the base, from which he rises on soiled pinion, to take a fancy sketch of the etherial nature of the vital principle; and most sagely concludes that as we cannot "bottle up a specimen" of this, therefore it would be impious to inquire in what manner inefficient pressure promotes the flow of blood from divided vessels. Well we cannot perceive the force of the argument or the legitimacy of the deduction, and do not feel inclined "to take off our shoes, and listen in reverence and in faith," because we do not believe that this constitutes any part of "the elementary teachings of the Great Master."

The analyst is now, in discharge of his professional duties, daily unfolding far more mysterious processes of Nature, and laboriously seeking to penetrate their "rationale," without any manifestation of that reverential awe which appears to have suddenly shed upon him its subduing and hallowing influence. Such a rhapsody from us, would probably have been treated as hypocritical cant—we regard it, in the analyst, as evidence of a pure and virtuous sentiment mis-applied. Such a limitation to philosophical investigation would, indeed, make short work of science, by dismissing all intermediate steps, and humbly referring everything to the final cause.

The gratuitous advice, of our self-constituted Mentor, we will take into grave consideration, and endeavor to profit by it. We are not old, we regret to say, we are not young, yet, such as we find ourself, we can say, in truth, that we are not inflated with any very exalted idea of our own ability or attainments—we know that we have none to boast of—and, therefore, we never exceed "a modest appreciation of our limited powers;" but, we are happily consoled by the reflection, that no extraordinary mental calibre is necessary to the successful execution of the self-imposed task of analysing the promised series of Surgical Essays, and we intend a faithful fulfilment of our purpose, unless prevented by more pressing and important engagements.

A "respectful consideration for the feelings" of Professor D. shall preside over every line we write, but we shall no more respect erroneous "opinions from men who were old in experience and in reputation, if not in years, when he (ourself) was a puling suckling" than if they emanated from a tyro just emerged from the green room. Indeed, it would be a manifest dereliction if we did not deal more harshly with the former, since their influence is far greater than that of the latter.

The last sentence of the analyst's advisory paragraph is too appropriate to himself; we must, therefore, beg to commend it to his serious meditation. Let him look again at the scurrility with which his analysis abounds—the low and vulgar expressions—the indecent allusions, etc., then "purify his conscience, and temper his feelings for the stern judgment which he must soon submit to, before Him who teaches that charity and brotherly love are the first of christian virtues."

And now in return for the good advice so kindly tendered to us we beg to make an humble return, in kind. "Personal detraction" and villification are the weapons of low and vulgar minds, and are utterly unworthy the station you occupy, the talent you possess or the character you have heretofore sustained.

Facts and reason are the weapons of pure and cultivated intellects, and, when wielded in a just and righteous cause, never fail to force conviction and to ensure a complete triumph.

You are young and ardent; but time will teach you the vast difference between these two classes of weapons, and a little effort at self-control on your part, will enable you to use the one adroitly and effectively, and to reject the other with scorn and loathing; whilst experience will instruct you, that no sensible person will believe you possess the nobler if you persist in the use of the meaner.

One fact is worth a thousand terms of reproach, and a single good reason will weigh more with sensible and virtuous people than all the abusive words that mar the purity and beauty of our language.

Remember, that motives are hidden deep in the human breast, and whether just or vicious, He only, whose eye penetrates the heart, can divine. Take warning by the fact, which you cannot now but perceive clearly, that while you venture to misconstrue the motives of others, your own may be liable, with appearance of far greater probability, to the same uncharitable animadversion.

Learn, that perfection is denied to man—that to err is human, and fall not down before any mortal, uttering—thou art immaculate, thou art immaculate!

In conclusion, relying on the conscious rectitude of our purpose—the truth and force of our facts, and the precision and correctness of our deductions, we willingly obey the summons of our opponent "to the bar of public opinion, and await the verdict." C.

